# Exosomal microRNAs derived from mesenchymal stem cells: cell-to-cell messages

**DOI:** 10.1186/s12964-020-00650-6

**Published:** 2020-09-11

**Authors:** Kasra Asgarpour, Zahra Shojaei, Fatemeh Amiri, Jafar Ai, Maryam Mahjoubin-Tehran, Faezeh Ghasemi, Reza ArefNezhad, Michael R. Hamblin, Hamed Mirzaei

**Affiliations:** 1 Department of Medicine, University of Western Ontario, London, Ontario Canada; 2 School of Medicine, Shahid Beheshti University of Medical Sciences, Tehran, Iran; 3 School of Allied Medical Sciences, Hamadan University of Medical Sciences, Hamadan, Iran; 4 Department of Tissue Engineering and Applied Cell Sciences, School of Advanced Technologies in Medicine (SATM), Tehran University of Medical Sciences (TUMS), Tehran, Iran; 5 Student Research Committee, Mashhad University of Medical Sciences, Mashhad, Iran; 6 Department of Medical Biotechnology, Faculty of Medicine, Mashhad University of Medical Sciences, Mashhad, Iran; 7 Blood Transfusion Research Center, High Institute for Research and Education in Transfusion Medicine, Tehran, Iran; 8 Department of Anatomy, School of Medicine, Shiraz University of Medical Sciences, Shiraz, Iran; 9 Wellman Center for Photomedicine, Massachusetts General Hospital, Harvard Medical School, 40 Blossom Street, Boston, MA 02114 USA; 10 Research Center for Biochemistry and Nutrition in Metabolic Diseases, Institute for Basic Sciences, Kashan University of Medical Sciences, Kashan, IR Iran

**Keywords:** Mesenchymal stem cells, Exosomes, MicroRNA

## Abstract

Exosomes are extracellular vesicles characterized by their size, source, release mechanism and contents. MicroRNAs (miRNAs) are single stranded non-coding RNAs transcribed from DNA. Exosomes and miRNAs are widespread in eukaryotic cells, especially in mesenchymal stem cells (MSCs). MSCs are used for tissue regeneration, and also exert paracrine, anti-inflammatory and immunomodulatory effects. However, the use of MSCs is controversial, especially in the presence or after the remission of a tumor, due to their secretion of growth factors and their migration ability. Instead of intact MSCs, MSC-derived compartments or substances could be used as practical tools for diagnosis, follow up, management and monitoring of diseases. Herein, we discuss some aspects of exosomal miRNAs derived from MSCs in the progression, diagnosis and treatment of various diseases.

**Video Abstract**

**Video Abstract**

## Background

Extracellular vesicles were first called “platelet dust” approximately five decades ago [1]. Apoptotic bodies, microvesicles and exosomes are the main types of EVs. EVs are generally characterized by their size and mechanism of release [2–4]. The main parameter used to define/characterize exosomes is their size. Different size ranges from 30 to 120 nm have been reported for exosomes [5–8], however they are now usually defined as < 150 nm vesicles [4]. The biogenesis of exosomes involves several steps starting with the production of multivesicular bodies (MVBs) from the endosomal system, followed by fusion to the plasma membrane, and finally secretion to the extracellular space [3, 4, 9]. Several different molecules and proteins are involved in exosome production and secretion [10–12]. Much evidence exists for the important role of these vesicles in intercellular communications and their involvement with both pathological and physiological conditions [13, 14]. Exosomes have also been implicated in intracellular communication, angiogenesis, immune system modulation and metastasis progression in cancer [15–18]. The techniques used to isolate and enrich exosomes from biological samples include precipitation-ultracentrifugation and ultracentrifugation followed by differential gradient centrifugation [6, 19–21]. Many biomolecules including DNA, mRNAs, miRNAs, proteins, lipids (and other substances such as vitamins and trace elements) have been shown to be present inside exosomes as cargos [22–24].

Non-coding RNAs, such as short hairpin RNA (shRNA), small interfering RNA (siRNA) [25] and microRNA (miRNAs) have attracted much attention from scientists and biotechnologists in terms of gene regulation, gene silencing, gene therapy and personalized cancer therapy [25–27]. miRNAs can regulate the expression of multiple mRNAs [28, 29], and play an important role in many biological processes, contributing to cell-cell communication [30, 31] as well as influencing the progression of different diseases [32, 33]. miRNAs are short ( ~22 nucleotides) non-coding RNAs, which regulate gene expression at the post-transcriptional level by binding to the 3′-untranslated regions of target mRNAs [34]. miRNAs play roles in every cellular process and are crucial for animal development, cell differentiation and homeostasis [35]. miRNAs are secreted into the extracellular fluid and might be used as biomarkers for disease diagnosis [36]. Exosomes containing miRNAs are secreted from most eukaryotic cells, particularly including mesenchymal stem cells (MSCs).

MSCs are adherent spindle-shaped cells expressing different special surface markers [37–42]. Their properties include the ability to differentiate to several different cell types for tissue regeneration in several diseases and injuries. MSCs also exert paracrine effects, and are anti-inflammatory and immunomodulatory [43–46].. Currently, MSCs can be isolated from different sources and have been employed as therapeutic approaches to treat several diseases and injuries [43–51]. However, many studies have concluded that MSCs were not easily engrafted into the target tissue as had initially been proposed; therefore, their benefit in tissue repair may be more due to their transient effects on the surrounding tissues. Moreover, the role of MSCs in either the remission or promotion of tumors remains controversial [46, 50].

Understanding the complex interplay between MSC-derived mediators (including exosomes, microvesicles and miRNAs) could be used as novel tools for diagnosis, follow-up, management and monitoring of many diseases. This therapeutic use of MSC-derived agents might be more practical than attempting to repair damaged tissues by direct use of intact MSCs [52–54]. In the present review we discuss some aspects of exosomal miRNAs derived from MSCs in the progression, diagnosis and treatment of different diseases.

## Mesenchymal stem cells as therapeutic tools

MSCs are characterized by their potential for continuous self-renewal by asymmetric division, and their ability to differentiate into a range of mesenchymal lineage cells such as skeletal myocytes, adipocytes, tenocytes, chondrocytes, osteoblasts, and visceral mesodermal cells [2, 13, 55]. Moreover, a number of researchers have proposed that MSCs can not only differentiate into cells of the common mesodermal lineage, but they may also differentiate into cells from ectodermal and endodermal origins, for example, neurons [15, 56], hepatocytes [57, 58], and cardiomyocytes [20, 59]. Moreover, it has been showed that secretome of MSCs could be different according to their origin [60]. For example, hMSCs derived from bone marrow (BMSCs), adipose tissue (ASCs) and human umbilical cord perivascular cells (HUCPVCs) differed in their secretion of axon guidance, axon growth, neurotrophic, neurogenic, and neurodifferentiative proteins, as well as, proteins with neuroprotective actions against oxidative stress, apoptosis and excitotoxicity, which have been shown to be involved in several CNS disorders/injuries processes [60]. It has been shown that the miRNAs contained in MSC-derived exosomes have important roles in the differentiation of MSCs [61]. The miRNAs that regulate osteogenic differentiation could affect exosomal functions such as the “Wnt signalling pathway” and “endocytosis” [62]. The potential of MSCs to differentiate into cells from different lineages are routinely evaluated using in vitro protocols with a range of unique differentiation media, and this in vitro data encouraged additional research into the use of MSCs as a resource for tissue regeneration. Because of the absence of many definititive MSC markers in vivo, we lack knowledge in comparison with in-vitro characterization.

Researchers have proposed that MSCs can trans-differentiate and engraft in-vivo, to repair defects in cartilage, bones [22], neuronal systems [25, 26], myocardial tissues [23, 24], and hepatic tissue [57]. Nevertheless, even if real treatment benefits are obtained, whether or not these benefits are due to true long-term engraftment, or to temporary paracrine effects, or to immunomodulatory effects remains to be firmly established.

By analysis of the effects of MSC-exomiRNAs, some targeted genes were identified which contribute to specific pathways related to cell death and cell growth, and pathways related to fibrosis such as Wnt signaling, PDGF, and TGF-β [63]. In another study it was found that lineage-specific exosomes could promote the odontogenic differentiation of human dental pulp stem cells (DPSCs) through the TGFβ1/Smad signaling pathway via transfer of miRNAs such as miR-27a-5p [64].

In one study, MSCs were genetically marked with green fluorescent protein (GFP) and injected intravenously, and studied in terms of engraftment and differentiation capacity [27]. GFP-marked MSCs were at first located in the lungs, and subsequently MSCs were found at low frequencies, in other organs, including hepatocytes, renal tubular cells, and bronchiolar epithelial cells.

**Fig. 1:**
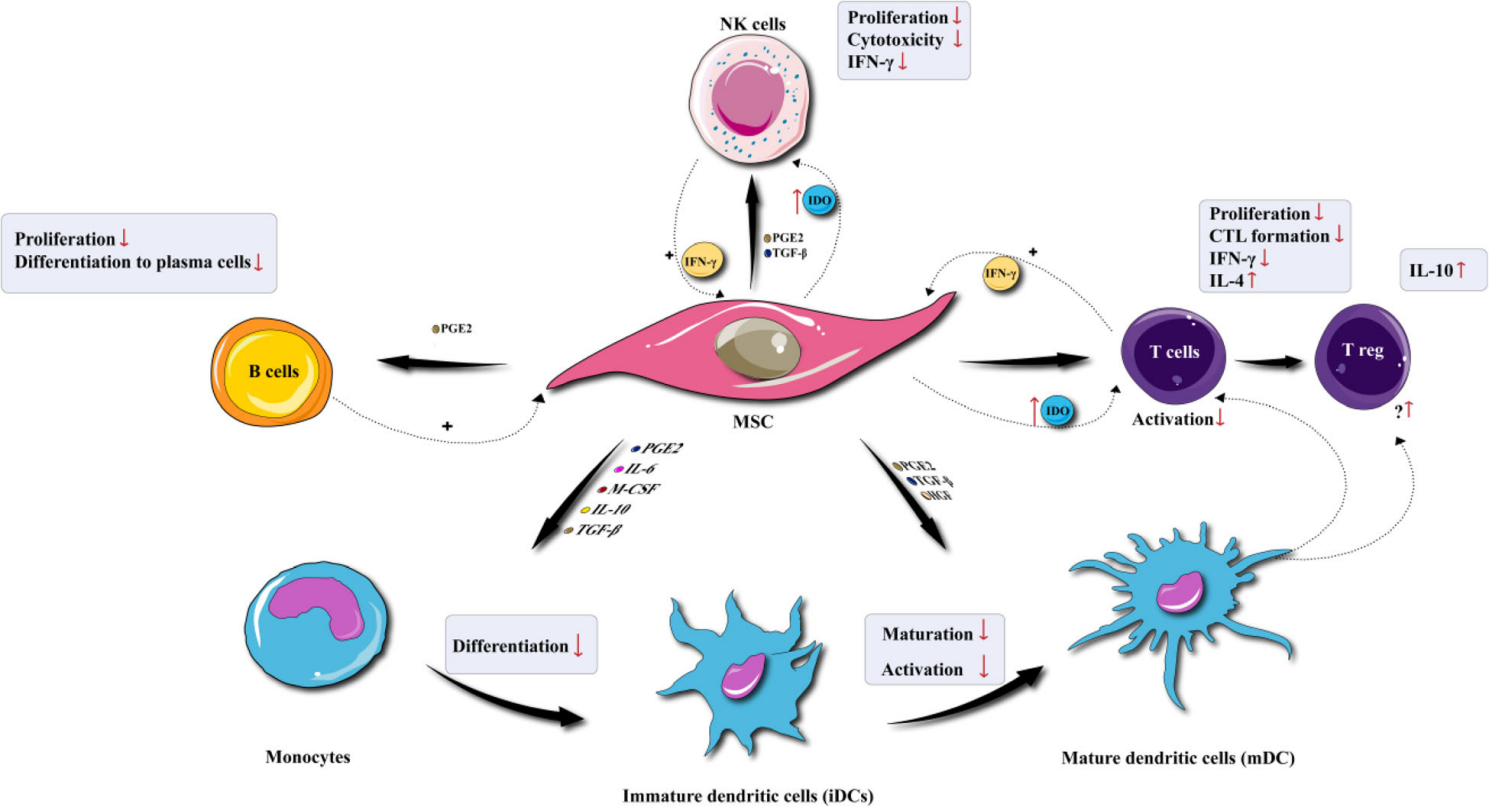
Immunomodulatory effects of MSCs. MSCs can affect immune system through increasing or decreasing the proliferation, differentiation, maturation, or activation of lymphocytes and other cells related to the adaptive and innate immune systems

Significantly, it has not yet been possible to document clonal expansion of the MSCs, and the benefits of MSCs in the regeneration of different tissues might result from other pathways. Most of the injected MSCs apparently eventually die off or undergo destruction inside the tissue. It is well-known that MSCs can carry out potent anti-inflammatory and immunosuppressive functions, and their important clinical benefits may be attributed to their effects on lymphocytes and other cells related to the adaptive and innate immune systems (Fig. 1). MSCs are known to suppress T-cell proliferation [30, 65, 66], B-cell activity [32, 66, 67], cytokine production, natural killer cell proliferation [68], and interfere with the function, differentiation and maturation of dendritic cells [28, 29, 33, 36, 69–72]. It has been demonstrated that MSC-ExomiRNAs such as miR-125a-3p can suppress the differentiation of effector T cells (CD4+ and CD8+), and improve the survival of CD4+, CD25+, Foxp3+ Tregs [73]. miR-146a down-regulates the NF-kB pathway involved in the inflammatory response [74]. Moreover, miR-21-5p, miR-142-3p, miR-223-3p, and miR-126-3p can attenuate the maturation and function of dendritic cells [75]. Indeed, miR-21-5p promotes the degradation of the C–C chemokine receptor type 7 (CCR7) gene, thus reducing the migration of DCs. Besides, miR-223-3p can prevent DC maturation, by acting on the CD83 gene. miR-126-3p targets tuberous sclerosis 1 (Tsc1), which is a negative regulator of mTOR kinase that in turn affects cell survival and proliferation. Furthermore, miR-142-3p can suppress the expression of the pro-inflammatory cytokine IL-6 [75].

Significantly, the expression of major histocompatibility complex (MHC) recognition and other co-stimulatory molecules on MSCs are attenuated [45]. The absence of MHC class II provides MSC with the potential to escape recognition by alloreactive CD4+ T cells. In addition to being MHC II negative, MSC do not appear to express the co-stimulatory molecules CD40, CD40L, CD80 or CD86 that are required for efficient destruction by effector T cells [76].

The early mechanism of MSCs may be due to the production of soluble factors, such as nitric oxide [47], indoleamine 2,3-dioxygenase (IDO) [48], hepatocyte growth factor (HGF) [65], and transforming growth factor-β [46]. Moreover they may also exert immunosuppressive functions through direct cell-to-cell contact. Additionally, MSCs may exert their immunosuppressive effect via increasing the number of regulatory T-cells (Tregs) by cell-to-cell contact and/or secretion of soluble agents [49, 77]. Moreover the increase in Tregs caused by MSCs in-vivo could be due to stimulating plasmacytoid dendritic cells to produce interleukin (IL)-10 [78]. These results suggest that MSCs can exert both direct immunosuppressive effects and indirect recruitment of Tregs for immune modulation.

The immunomodulatory effects of MSCs may be affected by the precise context of the inflammatory microenvironment. The immunosuppressive effects of MSCs will be increased by increased production of CXCL-10, IDO, CCL-8, and ICAM-1 [79–81]. Within severe inflammatory conditions, characterized by M1 macrophage polarization via and T-helper lymphocyte (Th)-type-1 cytokines, the pro-inflammatory cytokine interferon (IFN)-γ is predominant. MSCs tend to switch the polarization into M2 macrophages and Th2 cells for production of specific cytokines. This effect may explain why MSCs can encourage fibrotic processes [81], in situations of chronic inflammation. Hence, the therapeutic use of MSCs should be carefully considered, depending on the pertaining inflammatory microenvironment.

**Fig. 2:**
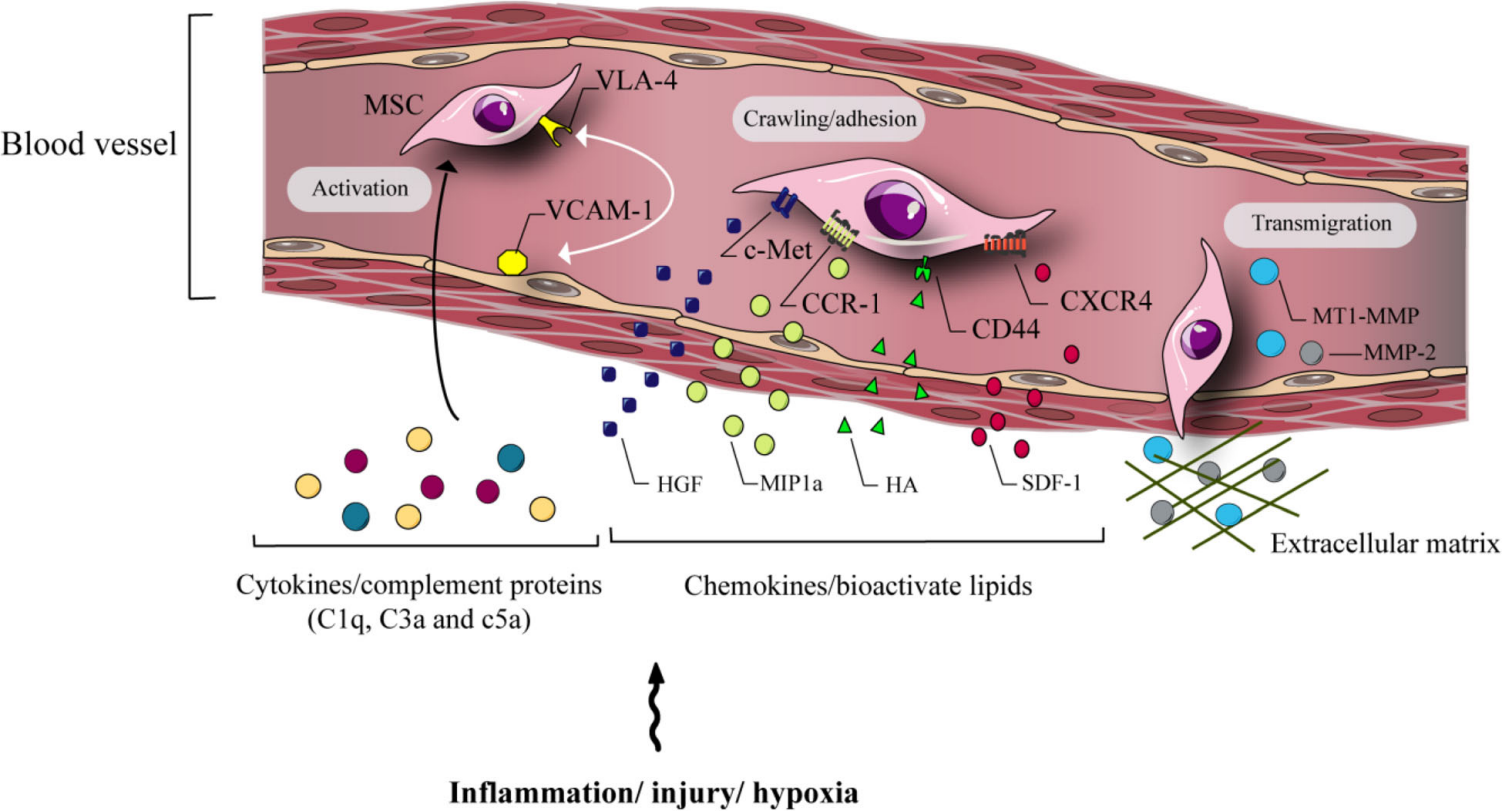
α4β1 integrin very late antigen-4 (VLA-4) is expressed by MSCs, and its respective ligand, vascular cell adhesion molecule (VCAM) isexpressed by endothelial cells. Inflammation in damaged tissues induces secretion of cytokines that up-regulate VCAM-1 and activate VLA-4, causing the binding of MSCs to the endothelial surface. Moreover, MSCs express various receptors, CD44, CXCR4, c-Met, CCR1 and respective ligands, such as M1P-1 (alpha), hyaluronic acid, SDF-1, and HGF, which are up-regulated in hypoxia and/or tissue damage. These ligand-receptor interactions, together with chemotactic bioactive lipids, regulate cell-to-cell contact between endothelial cells and MSCs. Complement proteins are induced in inflammation namely C3a, C1q, and C5a and also attract MSCs. MSCs express matrix metalloproteinases (MMP-2), and extracellular matrix-degrading membrane type (MT)1-MMP that can encourage extravasation

Some studies have suggested that MSCs have the innate ability to migrate to sites of inflammation and also to the tumor microenvironments. While all of the factors that contribute to MSC migration remain to be identified, research has suggested that MSC migration is caused by different chemokines and interplay with chemokine receptors, for example, stem cell factor (c-kit), HGF or c-Met [76], platelet derived growth factor (PDGF)/PDGF receptor [48, 49], vascular endothelial growth factor (VEGF)/VEGF receptor [77], monocyte chemo-attractant protein-1 (MCP-1), C-C chemokine Type 2 receptors [46], high mobility group box 1, receptor for glycation end-products [78, 79], stromal cell derived factor 1 (SDF-1), C-X-C chemokine Type 4 receptors (CXCR4) [47, 48], as well as additional cell adhesion molecules (Fig. 2) [47, 80].

Chemokines and cytokines and their receptors together play a critical function in leukocyte adhesion and migration, governing how leukocytes react to inflammation and tissue damage. Moreover, hematopoietic stem cells (HSC) likely also have roles in influencing MSCs. In addition, the tumor micro-environment behaves like an unhealed wound, constantly producing and secreting pro-inflammatory factors, chemokines, cytokines, and other chemo-attractant molecules [82]. Such continuous inflammatory signaling can be a trigger for MSC migration. CXCR4 and SDF-1 are critical factors for stem cell recruitment to tumors, via chemokine receptor signaling [83]. Moreover, the tumor microenvironment is characterized by hypoxia due to insufficient angiogenesis. The hypoxia-stimulated transcription factor HIF-1α triggers the transcriptional activity of many genes, comprising macrophage migration inhibitory factor (MIF), VEGF, tumor necrosis factor, and many pro-inflammatory cytokines [84]. For example, MCP-1 can encourage the migration of MSCs towards tumors [85].

Many receptors and chemokines have been implicated in stimulating the migration of MSCs, and more research is needed to distinguish between the factors, which stimulate MSC migration to tumors and to sites of inflammation [84]. The capacity of MSCs to migrate could facilitate the transfer of critical cargos such as ExomiRNAs to the target sites as well as transfer therapeutic miRNAs to damaged sites [52–54].

## MSCs-derived exosomes: an important biological molecule

A few years ago, MSC-derived exosomes were characterized in animal models. Depending on their particular origin, these EVs have some common properties, functions and applications, in spite of some differences in their surface marker profiles, their contents and size ranges. Due to the different origins of MSCs, their exosomes can express various markers including CD9, CD63 and CD81 [86, 87]. Some common MSC-derived exosomal CD markers, together with TSG101 and Hsp70 as purely exosomal markers have been used to characterize them [78, 80, 81]. On the other hand, some techniques, such as “NanoString” miRNA analysis, have been used to characterize miRNA sequences, and quantify miRNA expression levels in MSC-derived exosomes [79, 87]. Once exosomes released into the extracellular milieu can be taken up by target cells of the microenvironment or carried to distant sites through biological fluids, from which they have already been isolated, including urine, breast milk, blood, and cerebrospinal fluid [88].

MSC-derived exosomes have an important role in the ability of MSCs to function as stromal support cells to maintain homeostasis within the tissue, and respond to external stimuli, especially when the homeostasis of the microenvironment of the tissue is disrupted by disease or injury. MSC-derived exosomes are highly enriched in biologically active molecules, such as proteins and miRNAs, and are therefore well-equipped for this role [89].

A RNA profiling study of MSC-derived exosomes revealed that, along with miRNAs, MSCs exosomes were enriched in transfer RNAs (tRNAs) that can also function as miRNAs. Moreover, because the cellular expression profile of small RNA is not donor-specific or tissue-specific, the exosomal small RNA contents seem to be greatly influenced by the cell differentiation status [90]. The authors suggest that exosomal-contained small RNAs released by MSCs could control the microenvironment in the stem cell niche through maintaining a balance between differentiation and proliferation [90]. These findings may shed light on the regulatory mechanism of MSC paracrine activity responsible for the tissue-specific regenerative properties of MSCs.

Studies confirmed that of the MSCs engrafted at these sites of injury most MSCs are largely cleared, and only some of them do get through to the injured target tissue [91]. The tropism of MSCs is based on their inherent homing ability towards injury sites in response to factors and substances that are released from injured tissue [92]. In these tissues, MSCs release pro-angiogenic factors and EVs which contain functional molecules such as miRNAs. In this way, the local endothelium can be stimulated, and progenitor cells will differentiate into mature endothelial cells which can be incorporated into the injured tissue in order to promote vascular repair and regeneration [93].

The paradigm of MSCs-mediated regeneration has been shifted toward a secretome-based paracrine activity, rather than its cellular engraftment and differentiation. Indeed, it has been widely accepted that MSCs secrete bioactive factors with strong immunomodulatory activities. As a result, the use of MSCs conditioned medium has been put forward as a cell-free strategy with promising therapeutic effects. Initial studies from Timmers et al. (2007) on ischemia and reperfusion injury, demonstrated that only the fraction of the CM containing products > 1000 kDa provided cardioprotection [94].

**Fig. 3:**
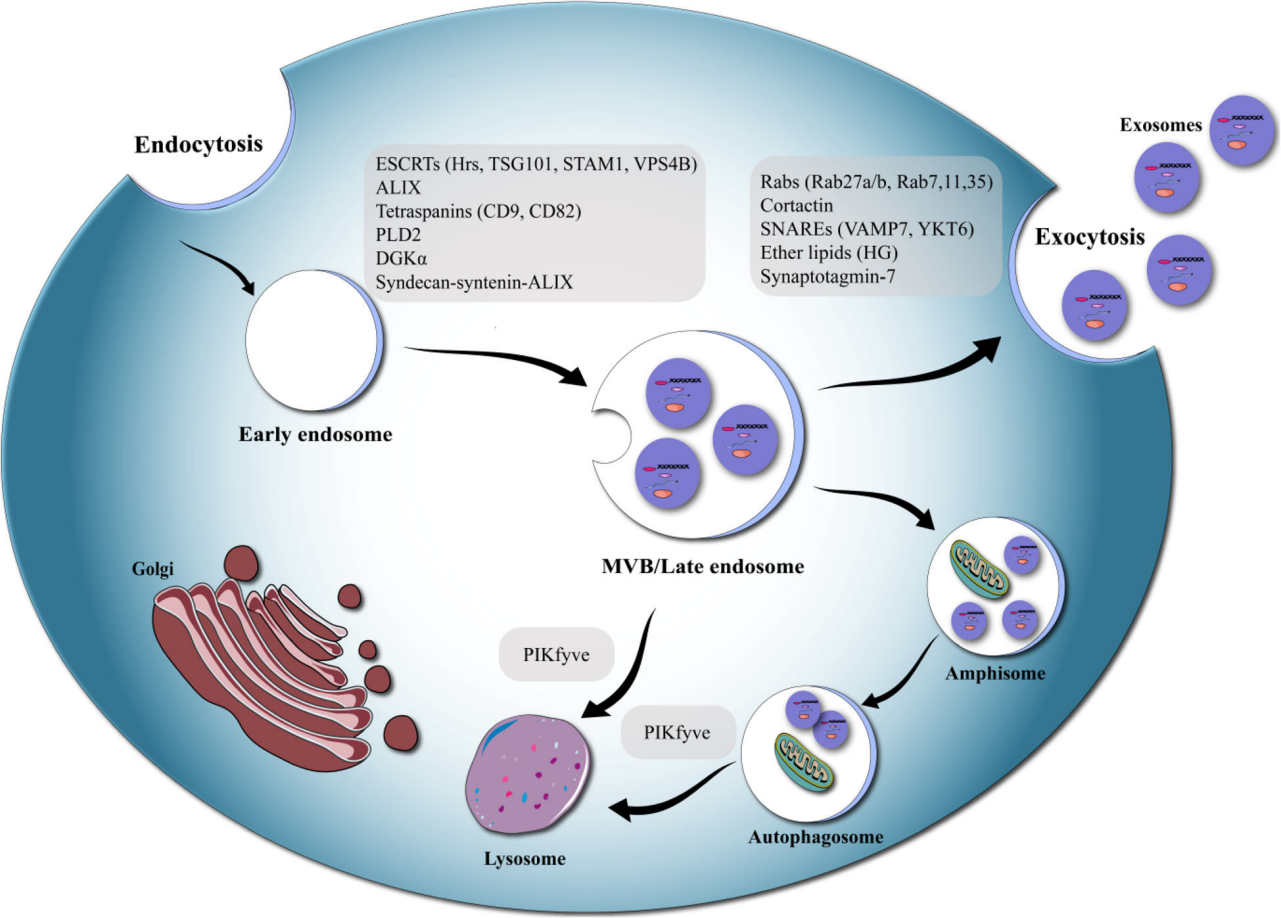
Exosome biogenesis. It has been demonstrated that many molecules influence exosome biogenesis and or release. The process of exosome secretion, may be divided into 3 steps: exosome biogenesis, transportation of MVBs to the plasma membrane, and fusion of MVBs with the plasma membranes

The biosynthesis of exosomes begins in the endosomal system. Early endosomes mature into late endosomes and in the course of this process, intraluminal vesicles ILVs are formed inside the multivesicular bodies (MVBs) (Fig. 3) [68]. If the MVBs fuse with the plasma membrane the ILVs can be released to the extracellular space. The ESCRT (endosomal sorting complexes required for transport) machinery is critical in this process. ESCRT contains 4 different proteins complexes; ESCRT-0, −I, −II, −III and the related AAA-ATPase Vps4 complex [28]. The most complete investigation of the role of ESCRTs in exosome biosynthesis used RNAi to target 23 ESCRT and ESCRT-related proteins in HeLa cells [29]. The released exosomes were captured on anti-CD63 beads and characterized using anti-CD81 and anti-HLA-DR (MHC II) antibodies and flow cytometry. After shRNA transfection, 7 ESCRT proteins, involved in exosome release were identified. Knockdown of TSG101, ESCRT-0, Hrs and the ESCRT-I protein STAM1 inhibited exosome release. On the other hand, knockdown of ESCRT-III and related proteins VPS4B, CHMP4C, ALIX, and VTA1 enhanced exosome release. Studies of the function of 4 proteins detected in exosomes separated by ultra-centrifugation, concluded that reduction of the levels of Hrs, TSG101, and STAM1 diminished exosome release, but reduction of VPS4B enhanced exosome release [29, 33, 36].

Knockdown of ALIX appeared to alter the protein composition of exosomes, rather than affecting exosome release. This suggested that ALIX had an effect on cargo loading, or the particular sub-types of MVBs, which were designated for release.

In three different studies, the ESCRT-0 protein Hrs was shown to be involved in exosome release [36, 72]. In the first study [33] reduction of Hrs in HEK293 cells was found to diminish release of exosomal Evi and Wnt3A [36]. In agreement, Hoshino and colleagues used Nanoparticle Tracking Analysis (NTA) to show that reduction of Hrs diminished exosome release from head and neck squamous carcinoma cells [72]. Membranes proteins bearing heparan sulfate chains, such as syndecans, contained inside syntenin–ALIX exosomes are regulated through their attachment to syntenin. Syntenin is a polyvalent soluble protein, which binds ALIX, hence, creating a connection between syndecans and the ESCRT machinery [71]. The interplay between syntenin and ALIX may govern the composition of ILVs [71]. The same group suggested that heparanases could trim the heparan sulfate side chains from syndecans, thereby encouraging binding to syntenin [70]. Importantly, the action of heparanase also showed the involvement of CD63 [70]. The syndecan–syntenin–ALIX pathway was responsible for about 50% of the released exosomes in MCF − 7 cells [69]. Other research has suggested that MVB biosynthesis may occur in cells lacking ESCRTs. Even with simultaneous knockdown of the sub-units of each of the 4 separate ESCRT complexes, ILVs were still produced in MVBs, showing the existence of an ESCRT-non-related mechanism [45]. Tetraspanins, are transmembrane proteins that are abundant in exosomes, especially in exosomes released by a ESCRT-unrelated pathway [46]. The enhancement of the exosomal release of β-catenin from HEK293 cells through the activity of tetraspanins CD9 and CD82 was suggested.

## MSCs-derived exosomes: as vehicle for crucial mediators

**Fig. 4:**
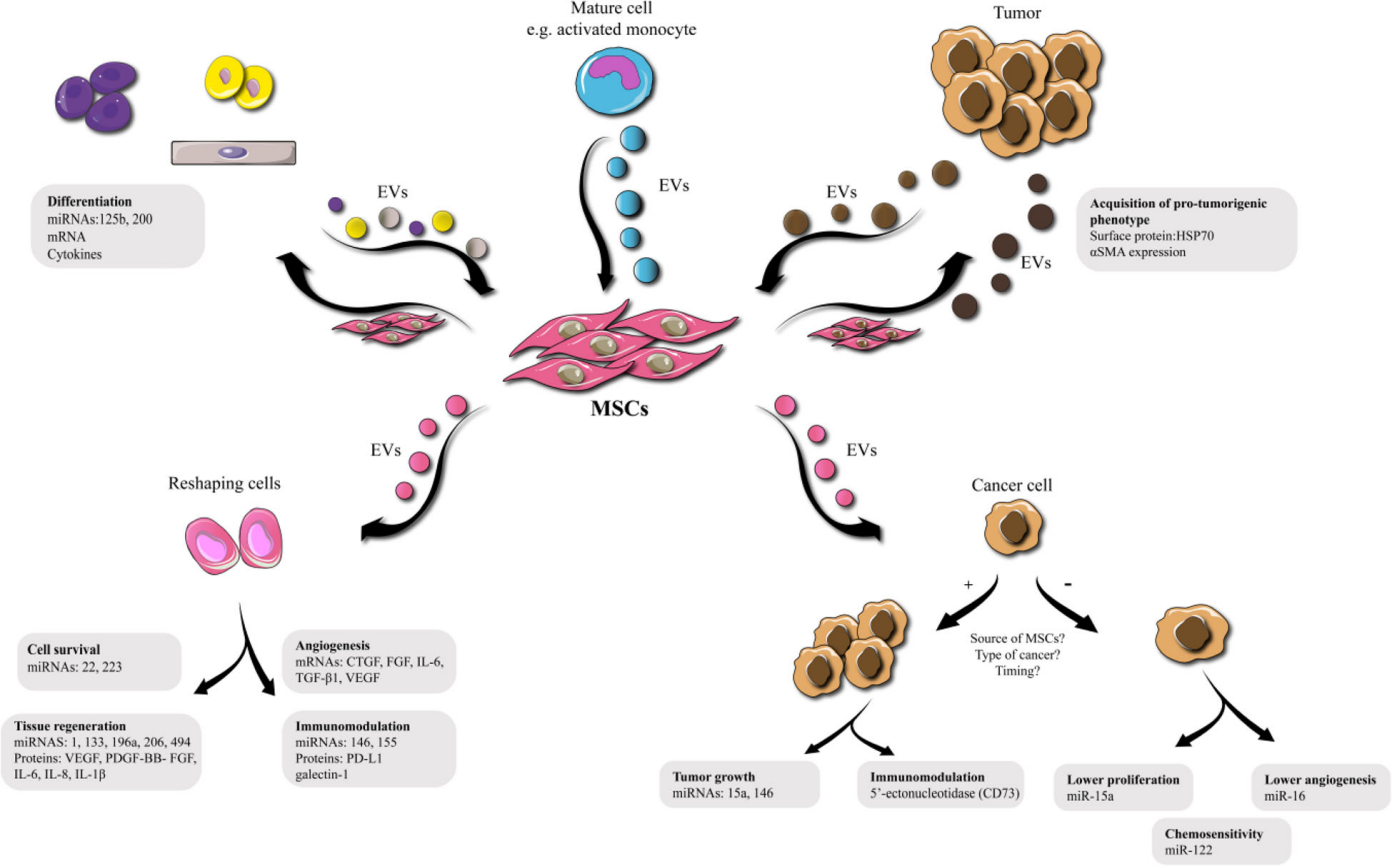
Inter-cellular communications between MSCs and other cell types via EVs**.** Upper panel: MSCs as the recipient cells. Lower panel: MSCs as the donor cells. Left Panel: MSCs exchange exosomes with recipient cells. Right Panel: Cross talk between MSCs and cancer cells. CTGF: connective tissues growth factor. EVs: extra-cellular vesicles. FGF: fibroblast growth factor. HSP: heat shock protein. IL: interleukin. mRNA: messenger RNA. miRNA or miR: microRNA. MSC: mesenchymal stem cell. PDGF: platelet derived growth factor. PD-L1 programmed death-ligand-1. SMA: smooth muscle actin. TGF: transforming growth factor. VEGF: vascular endothelial growth factor

MSC exosomes contain a vast range of contents, including genetic material, lipids, proteins and even some trace elements, [22–24], suggesting they could be used as tools for the diagnosis and treatment of different diseases [95]. Their immunomodulatory effects in particular, could be taken advantage of for the treatment of different diseases [95–97]. On the other hand, MSC-exosomes can also be loaded with exogenous cargos, such as biomolecules or different drugs, and then used as drug delivery vehicles for therapy [52–54, 97–99]. Modification of MSC-derived exosomes and loading with the desired cargo can be performed either after isolation, or during exosomes biogenesis [100]. The cargos may be derived from endogenous or exogenous origins [101]. Figure 4 shows inter-cellular communications between MSCs and other cell types via EVs**.** Exosomal drug delivery systems may be more efficient in comparison with liposomal-based systems. The advantages of exosomes include, less trapping of the carriers in macrophages located in the liver and spleen, lower toxicity, and easier capacity for modification [100, 102]. Modifications can be designed on the surface of the exosomes and the contents tailored for targeted drug delivery [103]. Murine MSCs that were treated with paclitaxel in vitro secreted paclitaxel-loaded exosomes which showed better anti-proliferative effects against a pancreatic adenocarcinoma cell line in vitro, compared to paclitaxel alone [97]. Paclitaxel-loaded exosomes from MSCs not only reduced breast cancer cell viability in vitro, but also inhibited tumor growth in vivo [104]. Other drugs such as doxorubicin can be loaded into MSC-derived exosomes to enhance drug delivery and improve cancer therapy efficacy [105]. Munoz et al. reported that targeted delivery of anti-miRNA-9 via MSC exosomes to glioblastoma multiforme cells down-regulated multidrug resistance 1 genes and restored chemosensitivity to these cells [106]. MSC-exosomal delivery of miR-143 to osteosarcoma cells resulted in decreased migration ability compared to lipofectamine-mediated miRNA delivery [107]. CXCR4-enriched exosomes derived from rat MSCs enhanced vessel formation, VEGF expression and cardiac function in an animal model with induced myocardial infarction [108]. Administration of engineered MSC exosomes over-expressing the *Akt* gene in a rat model of myocardial infarction led to significant improvement in endothelial cell migration, proliferation, vessel formation and finally boosted cardiac function [109]. As mentioned above, modification of MSCs and their exosomes might be a valuable strategy to increase their therapeutic potential. Tamura et al. isolated exosomes from MSCs and incubated them with cationized pullulan to modify the surface ligands in order to bind to hepatocyte asialoglycoprotein receptors. They showed this strategy increased the in vitro internalization of the exosomes into HepG2 cells, and in vivo the exosomes targeted injured liver tissue [110]. Other studies looked at the role of MSC exosomal cargos (such as various cytokines) to address pathological problems such as bladder tumors and graft versus host disease [111, 112]. Delivery of miR-133b via MSC-derived exosomes in an animal model of stroke caused by middle cerebral artery occlusion, improved cellular repair, neurite branching and outgrowth in the brain [113].

TNF-related apoptosis-inducing ligand (TRAIL) induces apoptosis in cancer cells, and has attracted attention from cancer researchers. Enhanced delivery of TRAIL using MSC-derived exosomes can work, even in TRAIL-resistant cancer cells [114]. Transfer of TRAIL via MSC exosomes was more effective than administration of recombinant TRAIL for induction of apoptosis [114]. High expression levels of miR-494 in MSC exosomes allowed its delivery to damaged muscle tissue to improve histological regeneration and capillary density [87].

Table 1 highlights some studies on MSC-derived exosomes and their cargo for the treatment of various diseases.

**Table 1:** Exosomes derived from mesenchymal stem cells and the respective cargo

Cargo	Disease	Source (type of MScs)	Detection method	Mode (in vitro, in vivo, human)	Ref
Paclitaxel	Pancreatic adenocarcinoma	Mouse MSC	–	In vitro	[97]
Paclitaxel	Breast cancer	Human MSCs	IVIS Lumina III imaging system	In vitro, In vivo	[115]
anti-miR-9	Glioblastoma multiforme	Human bone marrow	Flow cytometry, Western blot	In vitro	[106]
miRNA-143	Osteosarcoma	Human bone marrow	–	In vitro	[107]
CXCR4	Myocardial Infarction	Rat bone marrow	Real-time QPCR	In vivo, In vitro	[108]
Akt, PDGF	Myocardial Infarction	Human umbilical cord MSCs	Western blot	In vivo	[109]
HLA-G, TGF- beta and IL-10	GVHD	Human bone marrow	Cytokine release assay	In vivo, In vitro	[112]
TRAIL	Different cancer cell lines	Human MSCs	Immunofluorescence staining	In vitro	[114]

## MSC-derived exosomal microRNAs: small molecules with big actions

More than 30 years ago, miRNAs were identified as 22-nucleotide single-stranded non-coding RNAs, and were then rapidly introduced in molecular biology and biotechnology [30, 66]. The canonical and non-canonical pathways are two main mechanisms for miRNA biogenesis [30, 67, 116]. Briefly, RNA polymerase II transcribes the miRNA nuclear genes to produce pri-miRNAs. This hairpin-structure pri-miRNA is subjected to enzymatic cleavage to generate a pre-miRNA. The pre-miRNA is transported from the nucleus to the cytoplasm and is loaded onto the RNA-induced silencing complex (RISC) to form miRISC [32]. miRISC binds to target mRNA via complementary base pairing, leading to mRNA degradation and cleavage, finally resulting in post-translational gene regulation and/or silencing in higher eukaryotes [32, 68].

Because of the ability to transfer the contents of MSC-derived exosomes (especially their miRNAs) to neighboring cells or to enable their circulation around the human body, MSC exosomes could influence the pathogenesis and progression of diseases [117–120]. There have been reports implicating the role of MSC-derived exosomal miRNAs in the field of neurodegenerative disease, especially in Alzheimer’s disease [121], and also in stroke models. MSC-derived exosomal miRNAs could not only reduce apoptosis in damaged neural cells, but could also enhance brain function due to improving neuroplasticity [122–124]. Activation of the signaling pathway, PI3K/Akt/mTOR, mediated by the miR-17-92 cluster contained in MSC exosomes resulted in neuronal remodeling and neurogenesis in rodent models of cerebral stroke [125]. In another study, the authors carried out lentiviral transduction of miR-133b into rat MSCs and then isolated their exosomes. Their results showed that neural plasticity and functional recovery in a rat stroke model were improved following injection of these isolated exosomes [126].

Cancer cells can secrete exosomes as well as normal cells. Exosomal-packed miRNAs, including those secreted from MSCs, can influence cancer progression [127, 128]. MSC exosomes containing miR-21 and miR-34a were shown to promote tumor progression in renal and breast carcinoma [82, 129]. An enhanced level of miR-23b in bone marrow-derived MSC exosomes could alter the response of metastatic breast cancer to chemotherapy drugs, and decrease the invasive behavior [130]. Another study reported inhibition of in vitro angiogenesis via exosomal transfer of miR-100 with decreased levels of vascular endothelial growth factor [131]. Over-expression of miR-146b and miR-584-5p in transfected MSCs resulted in down-regulation of anti-apoptotic genes, up-regulation of pro-apoptotic genes and suppression of tumor growth in an in vivo malignant glioma model [92, 132].

No significant difference was observed between the immunosuppressive activities promoted by grafting of intact MSC and those stimulated by MSC-derived exosomes [133]. The possibility of allogeneic immune reactions in patients receiving MSC transplantation has hindered their use in tissue transplantation. The possibility of malignant alteration and uncontrolled differentiation are other problems that have been considered if MSCs were to be grafted into tissue and survive for the long term. However the use of MSC derivatives (rather than whole cells) may successfully overcome these problems [134]. Moreover, MSC-derived exosomes can be specifically designed to exert immunosuppressive effects through in vitro preconditioning of the MSCs before exosome isolation, thereby treating inflammatory and autoimmune conditions in a cell-free manner [134].

RGC (retinal ganglion cell) neuritogenesis and the survival rate were significantly enhanced by MSC-derived exosomes from bone marrow [135]. Mead and Tomarev demonstrated that the intravitreal application of MSC-derived exosomes considerably decreased the rate of loss of RGCs and increased RGC functional efficiency, utilizing a nerve crush model [135]. Injured RGCs were treated with MSC exosomes loaded with miR-17-92, miR-146 and miR-21 to evaluate the therapeutic potential. PTEN, a major RGC axonal growth inhibitor, was down-regulated by miR21 and miR-17-92, both of which were derived from MSCs. In addition, miR-146a, derived from the same source, suppressed EGFR (epidermal growth factor receptor) expression, a receptor which plays a part in the suppression of axonal regeneration. Interestingly, MSC-derived exosomes have been proposed to be therapeutically effective for enhancing the regeneration process of particular types of injured RGCs, because although MSC-exosomes could effectively promote regeneration and protection of RGCs in animal models, the result was not consistent with administration of exosomes sourced from fibroblasts [135]. Therefore, MSC-derived exosomes may be more clinically applicable for treatment of glaucoma, a condition characterized by a steady RGC decrease [136]. Direct inhibition of hepatocyte apoptosis has also been reported by MSC-exosomes, which were formerly shown to elicit immunosuppressive activity through suppression of natural killer cells [137, 138]. Administration of human menstrual blood-derived MSC-exosomes led to direct inhibition of caspase-3-mediated hepatocyte cell death in vitro. Moreover, this preparation also reduced acute liver injury promoted by lipopolysaccharide (d-GalN/LPS) and d-galactosamine in experimented mice, and reduced the mouse mortality rate [138]. The activity of MSC-EVs in inhibiting cell death was also supported by the findings of Chen et al., who worked on a murine model of autoimmune hepatitis (AIH) [137]. Mechanistically, this anti-apoptotic activity relied on the inhibition of NLRP3-mediated caspase-1 activation, and therefore reducing pyroptosis. The hallmarks of pyroptosis include the abundant secretion of IL-1β and IL-18 pro-inflammatory cytokines, osmotic lysis, DNA cleavage, cell swelling and further breakdown of the cytoplasmic membrane [139]. Unlike exosomes derived from MSCs without any miR-233, those with miR-233 successfully suppressed AIH. Therefore exosomal miR-233 can be considered to have a key role in the protective effect of these exosomes in the liver [137]. NLRP3 and caspase-1-dependent pyroptosis was down-regulated through MSC miR-233-stimulated degradation of hepatocyte NLRP3 mRNA, as shown by evaluation of the NLRP3-signaling pathway [137]. In addition, MSC exosomes were shown to be able to control hepatic inflammation induced by reactive oxygen species [140].

Intravenous injection of human umbilical cord MSC-derived exosomes led to improved renal cell function, and repair of an acute kidney injury in a rat model [141]. The authors reported higher expression levels of miR-15a, miR-15b and miR-16, and consequent lower expression of their target gene, CX3CL1. This means that these miRNAs as biological target molecules, by matching to the seed region of CX3CL1, may have the potential effects of modulation of CX3CL1 [141]. miRNA-30 containing human WJMSC-derived exosomes promoted renal function in an ischemia/ reperfusion injury model due to activation of DRP1 and consequent inhibition of mitochondrial fragmentation. It was found that the expression of miR-30 in injured kidney tissues was declined and mitochondrial dynamics turned to fission. Furthermore, when the miR-30 antagomirs were used to reduce the miRNA levels, all the related effects of EVs reduced remarkably. It also showed that a single administration of hWJMSC-EVs could protect the kidney from IRI by inhibition of mitochondrial fission via miR-30 [142]. Engineered MSCs that released miR-let7c containing exosomes, selectively migrated to the kidney in a model of TGF-β receptor 1 mediated fibrosis and improved the kidney function. The co-transfection of NRK52E cells using the 3′UTR of TGF-βR1 confirmed that miR-let7c reduced TGF-β1-driven TGF-βR1 gene expression. Therefore, the effective antifibrotic function of engineered MSCs is able to selectively transfer miR-let7c to damaged kidney cells and will pave the way for the use of MSC exosomes for therapeutic delivery of miRNA targeted at kidney disease [143].

Direct injection of MSC exosomes into the border region of a myocardial infarct decreased fibrosis and inflammation in an animal model [144]. Analysis of the target genes and pathways indicated that the PI3k-Akt-mTOR pathway could be main mechanism responsible for these phenomena because of up-regulation of miR-29, miR-24 expression, and down-regulation of miR-34, miR-130, miR-378 expression [144]. Enhanced expression of miR-29 prevented the development of kidney fibrosis by suppressing the synthesis of collagen [145]. Up-regulation of miR-24 limited aortic vascular inflammation. Importantly, in vivo expression of miR-24 in a mouse myocardial infarction model inhibited cardiomyocyte apoptosis, attenuated infarct size, and reduced cardiac death [146, 147]. Inhibition of miR-34 expression in vivo using LNA-based anti-miRs or antagomiRs improved cardiomyocyte survival after MI and thereby preserved cardiac contractile function. High expression of the miR-130 and miR-378 caused K+ ion channel dysfunction in cardiac stem cells and cardiac hypertrophy. Therefore, increased expression of miR-29 and miR-24 and reduced expression of miR-34, miR-130 and miR-378 may be responsible for the beneficial effects exerted by MSC-Exo [144]. Different exosomal miRNAs such as miR-22, miR-19a, miR-223 and miR-132 have been reported to exert cardioprotective effects by different authors, who proposed various related genes and signaling pathways as mechanistic explanations [40, 148–150].

MSCs and their associated mediators have attracted much attention for the treatment of liver diseases. Qu et al. transfected MSCs to over-express miRNA-181 and injected their isolated exosomes into a model of CCl4-induced liver fibrosis in mice. The mice showed amelioration of the liver fibrosis attributed to activation of the autophagy pathway. Exosomes from miR181-5p-ADSCs down-regulated Stat3 and Bcl-2 and activated autophagy in the HST -T6 cells. Furthermore, the up-regulated expression of fibrotic genes in HST-T6 cells induced by TGF -β1 was repressed following the addition of isolated miR181-5p-ADSC exosomes compared with miR-67-ADSC exosomes. Exosome therapy attenuated the liver injury and significantly down-regulated collagen I, vimentin, α-SMA and fibronectin in the liver, compared with controls. Taken together, the anti-fibrotic function of engineered ADSCs may be due to selective transfer miR-181-5p to damaged liver cells, and could pave the way for the use of exosome-ADSCs in liver disease [151].. Enhanced in vitro chemosensitivity of hepatocellular carcinoma cells was reported after a combination treatment with MSC exosomes containing miR-122 and chemotherapy agents. Tumor growth decreased in a nude mouse model of hepatocellular carcinoma after injection of these exosomes. This study suggested a novel strategy for increasing the chemosensitivity of HCC using AMSC-exosome-mediated transfer of therapeutic miR-122. The miR-122-modified AMSCs could effectively package miR-122 into the secreted exosomes, which mediated miR-122 transfer between the AMSCs and the HCC cells, thus further increasing the sensitivity of HCC cells to chemotherapeutic drugs through alteration of miR-122-target gene expression in these cells. Among the predicted targets of miR-122, ADAM10, IGF1R, and CCNG1 play key roles in tumorigenesis and drug sensitivity in various cancers [152]. An in vivo study in mice with induced autoimmune hepatitis treated with MSC exosomes over-expressing miR-223, found restoration of liver tissue due to reduced inflammation. Both BMSC-exosomes and BMSC-exosomes containing miR − 223 significantly reversed either S100 or LPS/ATP induced injury in mice and hepatocytes. Meanwhile, the expressions of cytokines, NLRP3 and caspase-1 were also down-regulated by BMSC-exosomes and BMSC-exosomes containing miR − 223 at both protein and mRNA levels in mice and hepatocytes. In conclusion, BMSC-derived exosomes could protect against liver injury in an experimental model of autoimmune hepatitis and the mechanism could be related to exosomal miR-223 regulation of NLRP3 and caspase-1 [137]. miR-122-modified MSC exosomes, not only reduced cell proliferation and collagen maturation in hepatic stellate cells in vitro, but also repaired the fibrotic liver via reduction of collagen deposition in the in vivo liver tissue [153].

There are several other studies reporting the roles of MSC-derived exosomal miRNAs in the management or manipulation of different physiological processes and pathological conditions/diseases including wound healing, inflammation, fracture healing, and Alzheimer’s disease [62, 96, 154–157]. Table 2 summarizes the findings of different studies regarding MSC exosomes and miRNAs.

**Table 2:** Exosomal microRNAs derived from mesenchymal stem cells and different pathological conditions

Disease	MicroRNA	Source (type of MSCs)	Target	Function	Model (In vitro, in vivo, human)	Ref
Intervertebral disc degeneration	miR-21	Human bone marrow	TNF-α	Apoptosis	In vivo, Rat	[122]
Stroke	miR-133b	Rat bone marrow	b-III-tubulin (TuJ1), phosphorylated NF-H RhoA	Cell shape, polarity and locomotion	In vivo, Rat	[123]
Stroke	miR-17-92 cluster	Rat bone marrow	PTEN	Tumor suppression	In vivo, Rat	[125]
Stroke	miR-133b	Rat bone marrow	Connective tissue growth factor and RhoA	Cell adhesion & migration. GTPase, actin stress	In vivo, Rat	[126]
Breast cancer and Osteosarcoma	miR-21 andmiR-34a	Human bone marrow	–	Increased angiogenesis & tumor growth	In-vivo, mice	[82]
Breast cancer	miR-23b	Human bone marrow	MARCKS	Cell cycling and motility	In vitro	[130]
Breast cancer	miR-100	Human bone marrow	Vascular endothelial growth factor (VEGF)	Angiogenesis	In vitro	[131]
Glioblastoma	miR-146b	Rat	EGFR	Cell proliferation and cell survival.	In vivo, Rat	[132]
Glioma	miR-584-5p	Human	CYP2J2	Arachidonic acid and drug metabolism	In vivo, Mice	[92]
Ischemia/reperfusion injury (IRI) -induced AKI	miR-15a, miR-15b and miR-16	Human umbilical cord	CX3CL1	Function as chemokine ligand	In vivo, Rat	[141]
Ischemia/reperfusion injury (IRI) -induced AKI	miR-30	Human WJMSCs	DRP1	Mitochondrial and peroxisomal division	In vivo, Rat	[142]
Kidney fibrosis and injery	miR-let7	Human Bone marrow	TGF-β receptor 1	Cell proliferation and apoptosis control	In vivo, Mice	[143]
Myocardial infarction	miR29, miR24miR34, miR130, miR378	Rat Bone marrow	PI3k-Akt and mTOR pathways	Cell cycle, proliferation and differentiation	In vivo, Rat	[144]
Myocardial infarction	miR-22	Ischemic Preconditioned Mice bone marrow	Mecp2, methyl CpG binding protein 2	Apoptosis	In vivo, mice	[148]
Myocardial infarction	miR-19a	Rat Bone marrow	PTEN	Tumor suppression,	In vivo, Rat	[149]
Myocardial infarction	miR-132	Mice bone marrow	RASA1	Cell proliferation and growth	In vivo, mice	[40]
Liver fibrosis	miR-181-5p	Mice adipose	Bcl2 and stat3	Apoptosis and autophagy	In vivo, mice	[151]
Hepatocellular carcinoma	miR-122	Human adipose	Cyclin G1 and IGF 1R	Cell cycle and growth	In vitro, and In vivo, mice	[152]
Autoimmune hepatitis	miR-223	Mice bone marrow	NLRP3	Cryopyrin production	In vivo, mice	[137]
Liver fibrosis	miR-122	Mice and human bone marrow	Cyclin G1, IGF 1R and P4HA1	Cell cycle and proliferation Collagen and related protein synthesis	In vitro, and In vivo, mice	[153]
Cardiotoxin-induced muscle injury	miR-494	Human bone marrow	–	Induce muscle regeneration via increased angiogenesis and myogenesis	In vivo, In vitro	[87]
Middle cerebral artery occlusion	miR-133b	Rat bone marrow	–	Modulate neurite outgrowth	In vivo, In vitro	[113]
Osteoarthritis	miR-92a-3p	MSCs	WNT5A	Enhanced production of cartilage and homeostasis	In vivo	[158]
Osteoarthritis	miR-320c	hBMSCs	MMP13	–	In vivo	[159]
Osteoarthritis	miR-140-5p	SMSC		Enhanced proliferation and migration of articular chondrocytes	In vitro	[160]

## Conclusion and future perspectives

MSCs are pleiotropic cells with the potential for migration and differentiation to repair tissue, but also possess significant paracrine effects and immunomodulatory potential. Despite many years of testing showing that the injection of MSCs can repair many different types of tissue damage and defects, the permanent engraftment of MSCs into tissue has been difficult to demonstrate. Therefore more attention is being paid to the ability of MSCs to affect neighboring cells in a beneficial but temporary manner. While directly secreted cytokines, chemokines, growth factors and other signaling molecules are undoubtedly important in the paracrine function of MSCs, exosomes are increasingly being investigated as vehicles by which biologically active mediators, especially miRNAs, can be transferred from MSCs to recipient cells. Exosomes derived from MSCs have been shown to possess potential benefits for the management of pathological conditions, and the possible cure of diseases. MSC-derived exosomes possess almost all of the properties of the original cells, in terms of paracrine effects and immunomodulatory functions. Moreover, these exosomes also possess the ability to accumulate at sites of tissue damage, without the ability to actively migrate by following chemokine gradients, as do their parent MSCs. MSC exosomes may also be ideal tools for drug delivery. Recently, loading MSC-exosomes with defined cargos using simple and versatile methods, has been suggested to be a promising strategy for treatment of different diseases. There are now many studies suggesting that MSC exosomes can be applied for cancer therapy, gene therapy, drug delivery, regenerative medicine and some other biomedical applications. Because of some limitations with the application of MSCs themselves, including controversial use in the presence of tumors, MSC-derived exosomes could be considered to be a cell-free alternative to intact MSCs.

## Data Availability

The primary data for this study is available from the authors on request.

## Abbreviations

BMDCs: Bone marrow dendritic cells
CXCR4: Chemokines receptors Type 4
DGKα: Diacylglycerol kinase α
EVs: Extracellular vesicles
IDO: Indoleamine 2,3-dioxygenase
MSCs: Mesenchymal stem cells
MVBs: Multivesicularbodies
shRNA: Short hairpin RNA
siRNAs: Small interfering RNA
GFP: Green fluorescent protein
MCP1: Monocytes chemo-attractant protein-1
MMP: Matrix metalloproteinases
MHC: Major histocompatibility complex
MLP-1: Latent membranes protein 1
nSMase2: Neutral sphingomyelinase 2
PDGF: Platelet derived growth factors
PLD2: Phospholipase D2
PLP: Proteolipid protein
HSC: Hematopoietic stem cell
VCAM: Vascular cell adhesion molecule
VLA-4: Very late antigen-4
TRAIL: TNF-related apoptosis-inducing ligand
